# Intraoperative microelectrode recording during asleep deep brain stimulation of subthalamic nucleus for Parkinson Disease. A case series with systematic review of the literature

**DOI:** 10.1007/s10143-024-02563-1

**Published:** 2024-07-20

**Authors:** Alessandro Izzo, Carla Piano, Manuela D’Ercole, Quintino Giorgio D’Alessandris, Tommaso Tufo, Maria Filomena Fuggetta, Federica Figà, Renata Martinelli, Marco Obersnel, Francesco Pambianco, Francesco Bove, Valerio Perotti, Anna Rita Bentivoglio, Alessandro Olivi, Nicola Montano

**Affiliations:** 1 Department of Neurosurgery, Fondazione Policlinico Universitario Agostino Gemelli IRCCS, Largo A. Gemelli 8, Rome, 00168 Italy; 2 School of Medicine, Università Cattolica del Sacro Cuore, Largo A. Gemelli 8, Rome, 00168 Italy; 3 Department of Neurology, Fondazione Policlinico Universitario Agostino Gemelli IRCCS, Largo A. Gemelli 8, Rome, 00168 Italy; 4 Department of Anesthesiology, Fondazione Policlinico Universitario Agostino Gemelli IRCCS, Largo A. Gemelli 8, Rome, 00168 Italy

**Keywords:** Microelectrode recording, Deep brain stimulation, Asleep, Parkinson disease

## Abstract

The use of microelectrode recording (MER) during deep brain stimulation (DBS) for Parkinson Disease is controversial. Furthermore, in asleep DBS anesthesia can impair the ability to record single-cell electric activity.

The purpose of this study was to describe our surgical and anesthesiologic protocol for MER assessment during asleep subthalamic nucleus (STN) DBS and to put our findings in the context of a systematic review of the literature. Sixty-three STN electrodes were implanted in 32 patients under general anesthesia. A frameless technique using O-Arm scanning was adopted in all cases. Total intravenous anesthesia, monitored with bispectral index, was administered using a target controlled infusion of both propofol and remifentanil. A systematic review of the literature with metanalysis on MER in asleep *vs* awake STN DBS for Parkinson Disease was performed. In our series, MER could be reliably recorded in all cases, impacting profoundly on electrode positioning: the final position was located within 2 mm from the planned target only in 42.9% cases. Depth modification > 2 mm was necessary in 21 cases (33.3%), while in 15 cases (23.8%) a different track was used. At 1-year follow-up we observed a significant reduction in LEDD, UPDRS Part III score off-medications, and UPDRS Part III score on medications, as compared to baseline. The systematic review of the literature yielded 23 papers; adding the cases here reported, overall 1258 asleep DBS cases using MER are described. This technique was safe and effective: metanalysis showed similar, if not better, outcome of asleep *vs* awake patients operated using MER. MER are a useful and reliable tool during asleep STN DBS, leading to a fine tuning of electrode position in the majority of cases. Collaboration between neurosurgeon, neurophysiologist and neuroanesthesiologist is crucial, since slight modifications of sedation level can impact profoundly on MER reliability.

**Supplementary Information:**

The online version contains supplementary material available at 10.1007/s10143-024-02563-1.

## Introduction

Microelectrode recording (MER), together with microstimulation, has been a mainstay for electrode positioning in subthalamic nucleus (STN) deep brain stimulation (DBS) for Parkinson Disease (PD) [35]. The recent improvement in targeting techniques and MRI have questioned its actual role [30]. A traditional limitation of MER was the need for awake surgery [35]. Differently from evoked potentials, which involve activation of groups of cells and bundles and therefore are easily recordable during general anesthesia, MER arise from single-cell registration [2] and thus are very susceptible to variations in anesthesia level. However, recent refinements in anesthesiologic technique permit to register MER also during general anesthesia [30, 32]. Whether the adoption of MER during asleep procedures results in some surgical advantage remains controversial.

In the present work, we described our surgical and anesthesiologic protocol allowing reliable MER recording during asleep STN DBS for PD, and we put our findings in the context of the available literature.

## Materials & methods

### Patients selection

This part of the report was drafted according with the Surgical techniqUe rePorting chEcklist and standaRds (SUPER) guideline [34], as far as applicable.

From January 2019 to October 2022, out of 83 DBS procedures, 32 patients underwent STN DBS for PD under general anesthesia with MER at our Academic Referral Hospital. Patients had a diagnosis of PD according to the United Kingdom Parkinson’s Disease Brain Bank criteria and satisfied the inclusion and exclusion criteria proposed by the core assessment program for surgical interventional therapies in Parkinson’s disease panel (CAPSIT-PD) [6], and were selected for asleep surgery based on several criteria such as older age, clinical conditions hindering awake surgery, low patients’ compliance, presence of brain atrophy and psychological status unfit to awake surgery. The study was conducted according to the principles set forth in the Declaration of Helsinki and later amendments. All patients signed an informed consent form according to the research proposals of our Institution. Implant was monolateral in 1 case and bilateral in the remaining ones, thus 63 electrodes overall were implanted.

The following clinical parameters were collected, where available, before surgery and at 1 year follow-up after surgery: levodopa equivalent daily dose (LEDD); Unified Parkinson's Disease Rating Scale (UPDRS) Part III (motor) score off-medications; UPDRS Part III (motor) score on-medications. UPDRS Part III scores at 1-year follow-up were registered while on stimulation.

### Surgical technique

A frameless technique (Nexframe™, Medtronic Inc, Minneapolis, MN, USA), using LeadPoint microelectrodes was used in all cases. Quadripolar implanted electrodes were: Vercise™ (Boston Scientific, Marlborough, MA, USA) (43/63), 3389 lead (Medtronic) (2/63) and Abbott/St Jude directed 6172 (Abbott laboratories, Abbott Park, IL, USA) (18/63).

Preoperative surgical targeting and trajectory planning was performed on StealthStation Surgical Navigation System (S8 Medtronic), using 1.5 T (29/32) or 3 T (3/32) MRI performed less than 1 month before surgery. Electrodes were placed using a mixed-technique targeting, in which predefined stereotactic coordinates based on the Schaltenbrand-Wahren atlas (x: ± 12 mm; y: -4 mm; z = -4 mm, using the midcommissural point as reference) were adjusted based on direct STN visualization on 3D FLAIR MRI images. The precoronal entry point was chosen to obtain the safest surgical trajectory, avoiding sulci, vessels, intraventricular or sub-ependymal trajectories.

In the operating room, after orotracheal intubation, the patient was placed on a passive headrest attached to a radiolucent Mayfield clamp base unit. O-Arm (Medtronic) was used to allow fiducials-less registration with excellent accuracy [26] and to check for electrode positioning. To optimize skin incision, a provisionary preoperative entry point was marked on the scalp using electromagnetic navigation. Two C-shaped incisions or, preferably, one semi curved pre-coronal incision were designed. O-Arm Imaging System was then positioned and centering 2D scan gained. The patient was prepped, draped and, after skin incision, burr holes were made, the lead-anchoring system was positioned and the Nexframe base and reference arc were secured. A standard-mode O-arm scan was taken for registration, acquired images were merged with preoperative MRI and the surgical planning was aligned to target. Target depth was thus calculated and set on the microTargeting™ Drive System (FHC Inc, Bowdoin, ME, USA) positioning device. The microelectrode recording equipment was set up in order to register the electrical activity of individual neurons from targeted structures. After the appropriate depth and trace were defined, the four-contact intracranial lead were placed. The procedure was repeated in the contralateral side. A final O-Arm scan was acquired to confirm proper lead placement. All O-arm scans were performed minimizing radiation dose to patients, applying ALARA principle (as low as reasonably achievable). Of note, the absorbed radiation dose after O-arm scan is 3-to-fourfold lower than after a standard brain CT scan [14].

### Anesthesiologic technique

The anesthetic technique is based on TIVA (Total IntraVenous Anesthesia) with TCI (Target Controlled Infusion) of both propofol and remifentanil, and bilateral scalp block using a mepivacaine 2% and ropivacaine 7.5 mg/mL mixture. TCI modality allows for a more precise control over the effects of anesthetic drugs on neurophysiological parameters, facilitating MER recordings. The scalp block allows sparing of anesthetic drugs. All patients received oral 0.2 mg/kg midazolam 1 h before surgery. Anesthesia was induced with a loading dose of remifentanil 2–3 ng/ml in continuous infusion (Alaris PK pump, Becton, Dickinson and Co, Franklin Lakes, NJ, USA) based on Minto pharmacokinetic model, followed 5 min with 3–3.5 µg/ml propofol. The TCI system used for target-controlled delivery of propofol was set up on Alaris PK pump and was based on Schnider's pharmacokinetic model [24]. Endotracheal intubation was facilitated by 0.08 mg/kg vecuronium bromide; no further doses of muscle relaxants were administered. The lungs were mechanically ventilated with a 45% O2 mixture in air, to maintain end-tidal CO2 (ETCO2) concentrations at 30–35 mmHg during surgery. Anesthesia was maintained with remifentanil 4 ng/ml and propofol 2.5–3.0 μg/ml according to patients’ physiological parameters and BIS (Bispectral Index) monitoring, to obtain a constant level of anesthesia. In the event of signs of light anesthesia (increased heart rate or mean arterial pressure > 15% from baseline, BIS > 50–60), the infusion rate of remifentanil was increased to 5 ng/mL and propofol up to 4 µg/ml. During the recording of evoked potentials, no bolus drugs were administered. At the end of the surgical procedure, all patients awakened within 15–20 min after stopping the TIVA.

### MER recording technique

Microelectrode is first placed in the central channel of the multielectrode holder for MER registering. MER is performed in 1-mm steps starting at 8–10 mm above the planned target and the tracks analysis is evaluated on LeadPoint system. According with literature evidence, STN is neurophysiologically identified by a sudden increase in background noise level and by the presence of neurons with characteristic 25- to 45-Hz firing rates and irregular firing patterns, with tremor-related activity and modulated by passive movements. The ventral border of the STN is recognized by a decrease of background noise and a decrease of multi-unit activity [12]. Continuous collaboration between neurosurgeon, neurologist, anesthesiologist and neurophysiology technician is mandatory to obtain clearly interpretable, repeatable and noiseless traces, maintaining tolerable depth of anesthesia. After the optimal depth has been defined, O-Arm scan confirms microelectrode position. In case of unclear/substandard neural activities, we collect secondary tracks, using the most appropriate additional channel based on lead position on O-Arm scan.

### Systematic review of the literature

Literature review was conducted in agreement with the PRISMA (Preferred Reporting Items for Systematic Reviews and Meta-analyses) guidelines statement [20]. Different medical databases (PubMed, Scopus, Embase) were screened for eligible scientific reports. The string used for the search was “(((microelectrode) OR (MER) OR (lead) OR (track) OR (trace)) AND ((general anesthesia) OR (asleep) OR (propofol) OR (TIVA))) AND ((deep brain stimulation) OR (DBS) OR (Parkinson) OR (Parkinson's))”. Any possible combination or name variation was explored. Last search was launched on April 20, 2024. Two reviewers (Q.G.D., R.M.) independently screened the abstracts and the references lists. Any difference was solved by consensus with a third senior author (N.M.). No restrictions on date of publication were made. Studies published in languages other than English were excluded from full-text review. We excluded studies assessing exclusively awake DBS, studies focusing exclusively on diseases other than Parkinson disease, and studies on DBS conducted under general anesthesia without MER recording.

### Statistical analysis and metanalysis

Among studies included in the systematic review of the literature, comparative studies detailing outcome of PD patients undergoing DBS with intraoperative MER under general *vs* local anesthesia were included in the metanalysis. The following data were collected, where available: baseline variables (age, Hohen&Yahr stage, PD disease duration); number of MER tracks per patient; outcome variables (reduction of UPDRS Part III score off and on medication, LEDD reduction); surgical morbidity. Metanalysis was performed using the random effect model. Standardized mean difference (SMD) with 95% confidence interval (CI) was calculated for continuous variables, and odds ratio (OR) with 95% CI was calculated for dichotomous variables. For each variable, a forest plot and a funnel plot were drawn. Heterogeneity was quantified by calculating the I^2^ statistic and considering as significant *p* < 0.05 and I^2^ > 50%. Publication bias was assessed using Egger’s test.

Comparison of paired measurement of continuous variables was performed using the Wilcoxon test for paired samples. Comparison of continuous variables between groups was performed using the Mann–Whitney *U* test. A *p* value less than 0.05 was considered significant.

MedCalc ver 22.023 (MedCalc Software Ltd, Ostend, Belgium) was used for all analyses.

## Results

### Case series

Mean age of included patients was 60.3 ± 7.1 years; 18 (56.3%) were male and 14 (43.8%) female. Mean PD disease duration was 12.2 ± 4.9 years. Median Hoehn&Yahr stage was 3. Mean preoperative planned STN coordinates were: x: ( ±)10.82 ± 1.01 mm; y: -3.33 ± 1.10 mm; z: -6.93 ± 0.94 mm. The mean duration of surgical procedure was 228 ± 70 min. No surgical complications were detected.

**Fig. 1:**
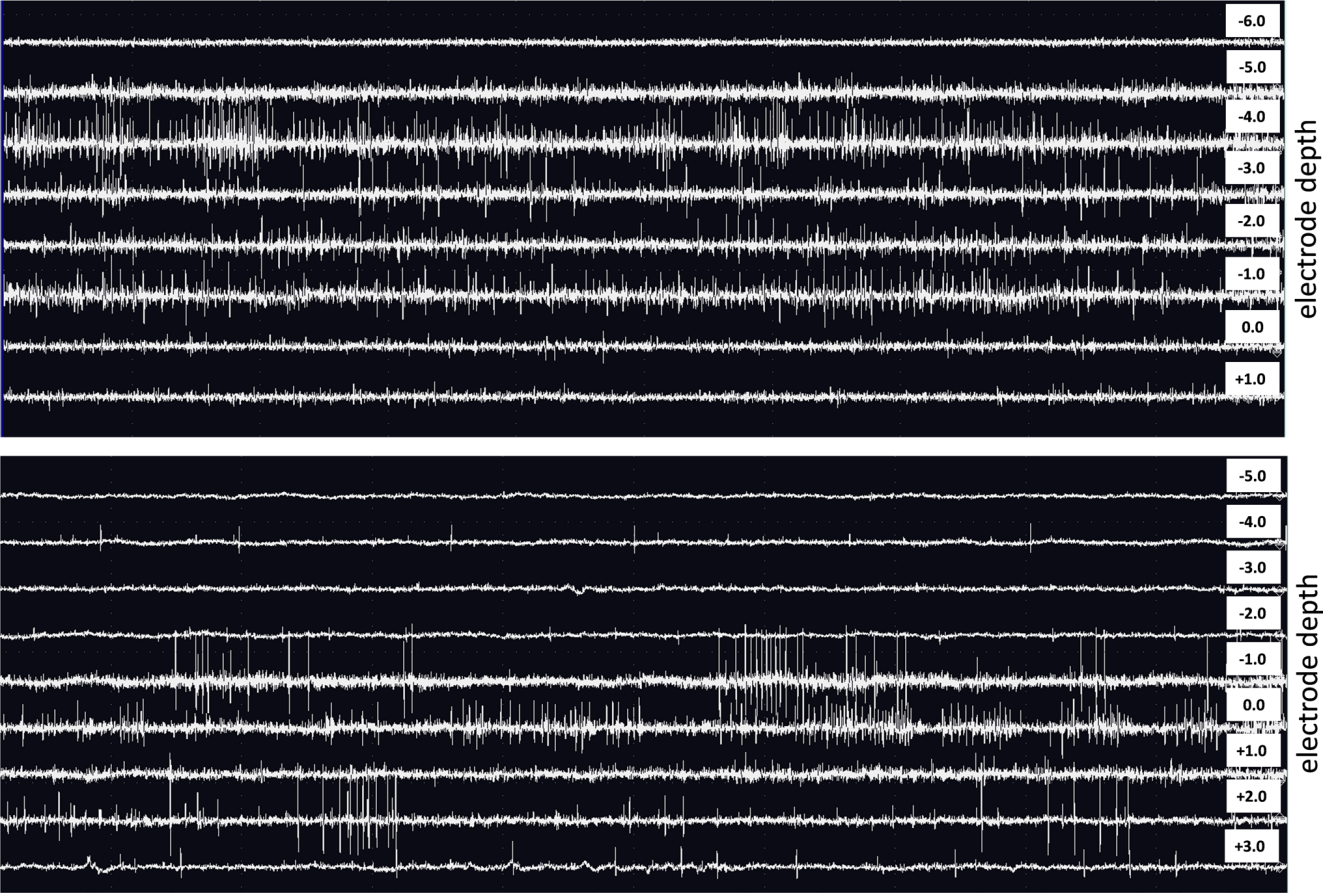
MER showing the typical STN discharge pattern in 2 PD patients operated under general anesthesia. Implanted depth, 0 *(upper patient)*, + 2 mm *(lower patient)*

MER could be reliably recorded in all cases (Fig. 1). Mean electrophysiological length of STN was 3.3 ± 1 mm on the right side and 3.4 ± 1 mm on the left side. Multiple tracks were explored using MER in 15 out of 63 sides (23.8%), leading to 81 overall MER registrations. In detail, two traces per side were explored in 12 cases, and 3 in 3 cases. The final electrode positioning based on MER recording is shown in Table 1. Overall, MER recording heavily influenced the final electrode positioning. In 2 cases only (3.2%) the planned target was adopted as the final lead tip position. Slight adjustments of the electrode depth (0.5–2 mm) were deemed necessary in 25 cases (39.7%). Most importantly, depth modification > 2 mm was performed in 21 cases (33.3%), while in 15 cases (23.8%) a change in trajectory was necessary. In detail, the lateral trajectory was chosen in 9 cases, the anterior one in 2, the medial one in 3, and the posterior one in 1 case.

**Table 1:** Detail on electrode placement

Electrode placement (mm from planned target)	*n*	%
0	2	3.2%
0.5	1	1.6%
1	8	12.7%
1.5	3	4.8%
2	13	20.6%
2.5	5	7.9%
3	10	15.9%
4	6	9.5%
Other tracks	15	23.8%
Total	**63**	**100.0%**

Clinical patients data are presented in Table 2. At 1-year follow-up, mean stimulation parameters were as follows: amplitude 2.4 ± 1.0 mA, pulse width 52.3 ± 12.3 ms, and frequency 142.2 ± 30.9 Hz. By comparing patients who intraoperatively had track modifications with those in which only the central MER track was used, a shorter pulse width in the former was recorded (46.1 ± 13.3 ms vs 57.3 ± 8.8 ms, *p* = 0.0034, Mann–Whitney *U* test). Importantly, at 1 year DBS determined a significant reduction in UPDRS Part III score off-medications (38% reduction from baseline), UPDRS Part III score on-medications (14% reduction from baseline), and LEDD (42% reduction from baseline), as compared to baseline (*p* = 0.0003, *p* = 0.0003 and *p* = 0.0376, respectively; Wilcoxon test for paired samples). Improvement was not different comparing patients who intraoperatively had track modifications with those in which only the central MER track was used (*p* = 0.5964, *p* = 0.3850 and *p* = 0.1388 for UPDRS part III score off and on medications and for LEDD, respectively; Mann–Whitney *U* test). As concerns side effects, we recorded one case of dysarthria, one seizure after the start of the stimulation, and 2 cases of dystonia. All these effect were successfully managed by adjusting stimulation parameters.

**Table 2:** Clinical data before surgery and at 1-year follow-up

Parameter	Mean preoperative value	Mean postoperative value	p*
LEDD	1221.8 ± 414.1	708.1 ± 288.3	0.0003
UPDRS Part III score off medication	42.6 ± 14.5	26.2 ± 8.7	0.0003
UPDRS Part III score on medication	19.8 ± 6.2	17.1 ± 9.0	0.0376

*LEDD* Levodopa equivalent daily dose; *UPDRS* Unified Parkinson's Disease Rating Scale

*Wilcoxon test for paired samples

### Systematic literature review and metanalysis

**Fig. 2:**
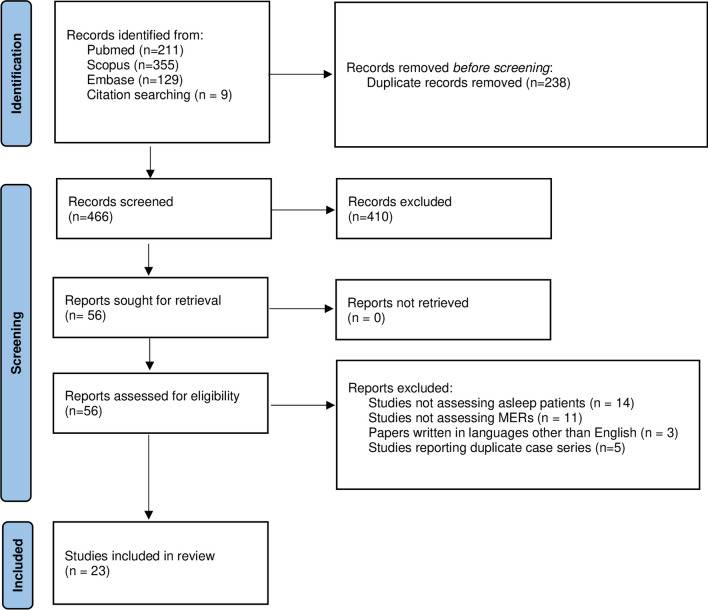
The PRISMA 2020 flow diagram

The search of the literature, after duplicate removal and citation searching, yielded a total of 466 results. After title and abstract screening, 56 studies were found to be relevant to the present study and thus included for full-text review. Upon full-text review, 33 studies were excluded because they described procedures not performed under general anesthesia (*n* = 14), not assessing MERs (*n* = 11), because reports were written in languages other than English (*n* = 3), or described cases already included in other papers by the same research groups (*n* = 5). Thus, 23 articles were included in the review. (Fig. 2 and Table 3) [1, 3–5, 8–11, 13, 15–19, 21, 23, 25, 27–29, 32, 33, 35].

**Table 3:** Systematic review of the literature describing successful MERs during asleep DBS surgery for Parkinson Disease

Author, Year	Type of study	Compa-rative study (awake vs. asleep)	*N* of asleep cases	Target	Frame/Frameless procedure		Targeting method	Anesthesia level monitoring (device)	Drugs used for GA	MER device	DBS success	MER Comments
Asha et al. 2018 [1]	Prospective	No	56	STN	Frame		Mixed	NA	Propofol	LeadPoint (Medtronic)	Significant clinical improvement	Study focused on DBS while on medications. Reliable MER under GA in patients on dopaminergic therapy
Blasberg et al. 2018 [3]*	Retrospective	Yes	48 (vs 48 awake)	STN	Frame		Mixed	NA	Propofol, Remifentanil	NA	Clinical improvement a 3 months more marked in awake group; no difference at 1 year follow-up	No significant difference in number of MER traces between awake and asleep patients
Chen et al. 2011 [4]*	Retrospective	Yes	33 (*vs* 19 awake)	STN	Frame		Direct	Yes	Desflurane	LeadPoint (Medtronic)	Motor improvement at 12 months in both groups without significant differences. Significant deterioration in cognitive function in the GA group	No significant differences regarding MER were observed between the GA and LA groups
Chen et al. 2021 [5]	Prospective	No	12	STN	NA		NA	Yes	Desflurane (6), Sevoflurane (6)	NA	Clinical improvement	Neuronal firing rate was lower with desflurane than with sevoflurane anesthesia. However, drug choice did not influence STN mapping accuracy or the clinical outcome of DBS electrode implantation
Fluchere et al. 2014 [8]	Prospective	No	213	STN	Frame		Mixed	Yes	“Controlled” GA with sevoflurane and alfentanil	LeadPoint (Medtronic)	Clinical improvement a 1 and 5 years	Use of halogenate gases allows to avoid propofol, which is deemed to modify brain electrical signals
Harries et al. 2012 [9]	Retrospective	No	82	STN	Frame		Direct	Yes	Nitrous oxide + isoflurane (26) Propofol + remifentanil (56)	LeadPoint (Medtronic)	Clinical improvement at 3, 6 and 12 months	Excellent quality of STN MER was obtained under general anesthesia (with no difference between the two types of GA used)
Hertel et al. 2006 [10]	Retrospective	No	9	STN	Frame		Indirect	Yes (BIS)	Propofol, remifentanil	LeadPoint (Medtronic)	Clinical improvement at 7 months	Typical STN bursting cells could be registered, but a significant widening of the background baseline noise could not be identified in the STN
Holewijn et al. 2021 [11]*	Prospective	Yes	54 (*vs* 56 awake)	STN	Frame		Direct	NA	Propofol, remifentanil	NA	Clinical improvement at 6 months in both groups without significant differences	NA
Jiang et al. 2021 [13]*	Retrospective	Yes	35 (*vs* 33 awake)	STN	Frame		Direct	Yes (Narcotrend monitoring, MonitorTechnik)	Propofol, remifentanil	LeadPoint (Medtronic)	Clinical improvement at 6 months in both groups without significant differences	No differences in MER between asleep and awake patients
Lefranc et al. 2017 [15]*	Retrospective	Yes	13 (*vs* 10 awake)	STN	Frame		Direct	Yes	“Controlled” GA with sevoflurane and alfentanil	NA	Clinical improvement at 12 months in both groups without significant differences	“Controlled” GA facilitates MER of STN neuronal activity
Lettieri et al. 2012 [16]	Retrospective	Yes	5	STN	Frame		Direct	NA	Remifentanil, ketamine	LeadPoint (Medtronic)	NA	Study describing re-implantation under GA of electrodes formerly implanted under LA. No differences in MER parameters between LA and GA procedure
Lin et al. 2020 [17]	Prospective	No	23	STN	Frame		Direct	Yes (BIS)	Propofol, dexmedetomidine	LeadPoint (Medtronic)	NA	Determination of the propofol dose able to inhibit MER recording
Lu et al. 2022 [18]*	Retrospective	Yes	76 (*vs* 81 awake)	STN	Frame		Mixed	Yes (BIS)	Propofol, remifentanil	NA	Clinical improvement in both groups	NA
Myrov et al. 2019 [19]	Retrospective	Yes	4 (*vs* 4 awake)	STN	Frame		Indirect	Yes (BIS)	Propofol	NA	NA	The Authors reported on differences in 25 MER parameters of STN single unit activity in awake *vs* asleep DBS. Fourteen parameters were influenced by GA; mainly, GA caused decrease in firing rate and a increase neuron bursting
Park et al. 2020 [21]*	Retrospective	Yes	90 (*vs* 57 awake)	STN	Frame		Direct	Yes (BIS)	Propofol, fentanyl	LeadPoint (Medtronic)	Clinical improvement in both groups. Greater LEDD reduction in GA group	NA
Qian et al. 2023 [23]*	Retrospective	Yes	22 (*vs* 18 awake)	STN	Frame		Direct	Yes (BIS)	Propofol, remifentanil	NA	Clinical improvement at 6 months in both groups without significant differences	MER signals under GA were interfered but clearly recognized. Similar outcome in GA and LA patients
Senemmar et al. 2021 [25]*	Retrospective	Yes	80 (*vs* 24 awake)	STN	Frame	Mixed		NA	Propofol, remifentanil	BenGun system	At 3 months therapeutic window was wider in the asleep group	MER feasible in asleep patients. Less MER traces in asleep
Tsai et al. 2016 [27]	Prospective	Yes	8 (*vs* 8 awake)	STN	Frame		Mixed	Yes	Desflurane	LeadPoint (Medtronic)	No difference in clinical outcome, stimulation parameters and adverse effects between GA and LA	Trend toward a lower STN firing rate under GA, without clinical impact
Tsai et al. 2020 [28]*	Retrospective	Yes	10 (*vs* 9 awake)	STN	Frame		Direct	Yes	Sevoflurane	LeadPoint (Medtronic)	Clinical improvement at 5 years in both groups without significant differences	STN firing properties and gray–white matter transitions readily identifiable under GA with sevoflurane
Vesper et al. 2022 [29]*	Retrospective	Yes	63 (*vs* 17 awake)	STN	Frame		Mixed	NA	Propofol, remifentanil	Inomed GmbH	Clinical improvement at 3 months in both groups	This report describes an anesthesia protocol that makes possible MER during asleep DBS
Wu et al. 2023 [32]	Retrospective	No	255	STN	Frame		Direct	NA	Propofol	NA	NA	Article focused on post-operative pneumocephalus. MER passages > 3 predicted larger pneumocephalus in patients with elevated head
Yamada et al. 2007 [33]*	Retrospective	Yes	15 (*vs* 10 awake)	STN	NA		Indirect	NA	Dinitrous oxide, sevoflurane, propofol and/or fentanyl	NA	Clinical improvement at 3 months in both groups without significant differences	NA
Zhao et al. 2022 [35]*	Retrospective	Yes	20 (*vs* 23 awake)	STN	Frame		Mixed	Yes (BIS)	Propofol, remifentanil	LeadPoint (Medtronic)	Clinical improvement with drug dose reduction at 6 months in both groups without significant differences	Even with BIS > 70, GA has a certain impact on intraoperative MERs, but the typical STN discharge is clearly identifiable
Present Study	Retrospective	No	32	STN	Frameless		Mixed	Yes (BIS)	Propofol, remifentanil	LeadPoint (Medtronic)	Clinical improvement at 1 year	MER are recordable reliably under GA and profoundly influence the final electrode positioning

*study included in the metanalysis. *BIS* Bispectral index; *GA* General anesthesia; *LA* Local anesthesia; *LEDD* Levodopa-equivalent daily dose; *MER* Microelectrode recording; *NA* Not available

Overall, including also the present series, the present review encompasses 1258 DBS cases for PD in which MER were performed under general anesthesia. In all cases, the target was the STN. Intriguingly, all reviewed studies used a frame-based procedure – ours is the only series adopting a frameless DBS technique. Fourteen papers describe some monitoring of anesthesia level, the most popular being BIS, which has been adopted also in our series. Regarding the drugs used for general anesthesia, 15/23 papers used a TIVA protocol, mainly based on propofol and remifentanil, which was the drugs combination used also in the present series. No papers report an unfavorable outcome of asleep patients operated using MER.

**Fig. 3:**
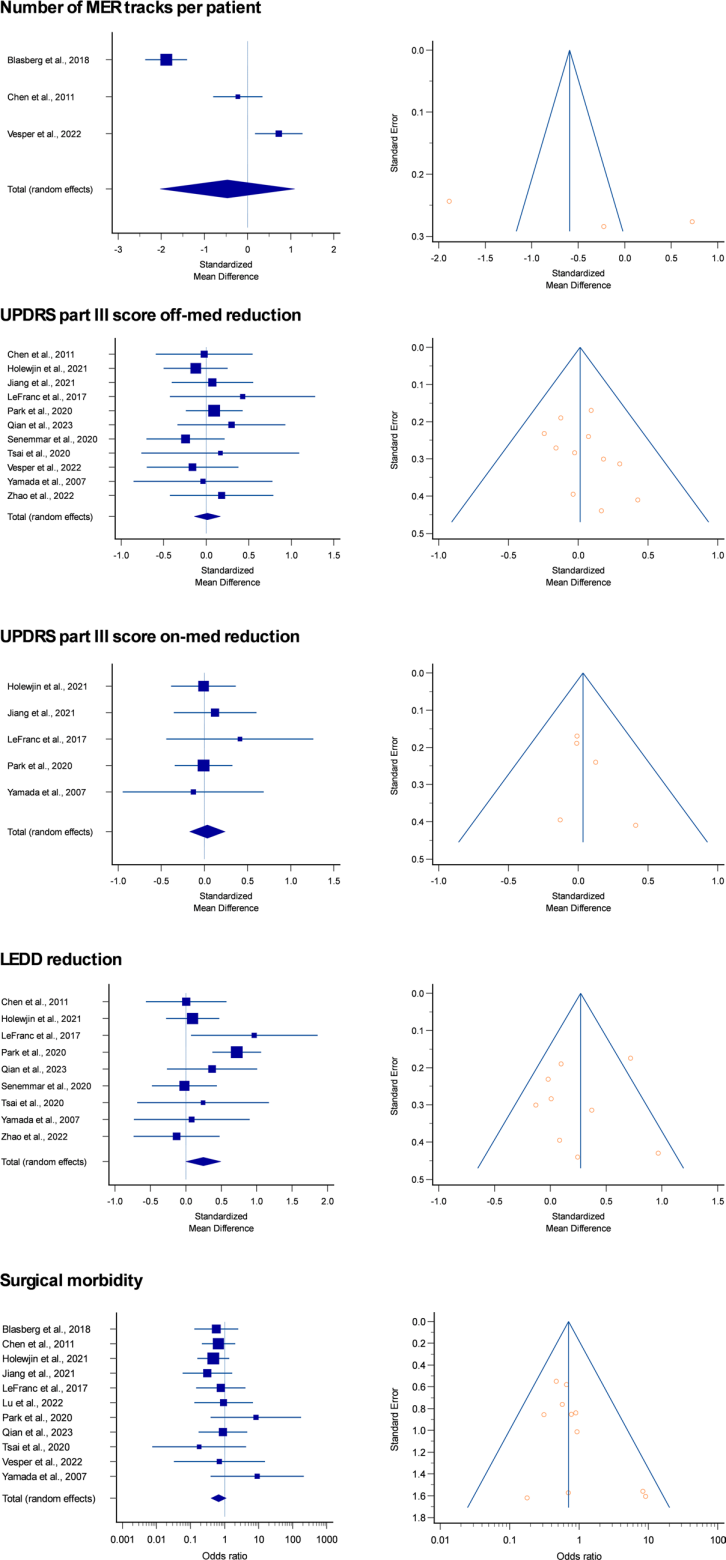
Forest plots (left column) and funnel plots (right column) regarding, from top to bottom, number of MER tracks per patient, reduction in UPDRS Part III score off and on medication, LEDD reduction and surgical morbidity. In each forest plot, right favors asleep DBS and left favors awake DBS

Thirteen studies were included in the metanalysis, whose results are shown in Table 4, Fig. 3 and Supplementary Fig. 1. Baseline parameters (age, Hohen&Yahr stage, PD disease duration) were similar between patients undergoing DBS with intraoperative MER under general *vs* local anesthesia (Table 4 and Supplementary Fig. 1). Number of MER tracks did not differ significantly between asleep and awake patients, nor significant differences in the improvement of UPDRS part III score off- or on-medications were demonstrated. A nonsignificant trend at greater LEDD reduction in asleep patients was evidenced. Finally, surgical complications rate was similar between the two groups (Table 4 and Fig. 3).

**Table 4:** Meta-analysis of studies comparing use of MERs during asleep *vs* awake DBS surgery for Parkinson Disease

Factor	OR/SMD	95% CI	*p*-value	I^2^	I^2^ *p*-value	Egger’s test *p*-value
Age	0.0382^#^	-0.124 to 0.200	0.643	18.33%	0.2690	0.4381
Hoen&Yahr Stage	0.000507^#^	-0.166 to 0.167	0.995	0%	0.9592	0.4831
PD disease duration	0.142^#^	-0.114 to 0.399	0.276	65.15%	0.0014	0.9181
N MER tracks per patient	-0.467^#^	-2.028 to 1.094	0.556	96.19%	< 0.0001	0.3222
UPDRS Part III score off medication reduction	0.0136^#^	-0.140 to 0.167	0.862	0%	0.9070	0.2440
UPDRS Part III score on medication reduction	0.0343^#^	-0.170 to 0.239	0.741	0%	0.8654	0.4254
LEDD reduction	0.247^#^	-0.000296 to 0.495	0.05	45.16%	0.0677	0.7164
Surgical morbidity	1.494*	0.897 to 2.489	0.123	0%	0.6791	0.1324

^#^SMD; *OR. *CI* Confidence interval; *LEDD* Levodopa-equivalent daily dose; *MER* Microelectrode recording; *OR* Odds ratio; *PD* Parkinson Disease; *SMD* Standardized mean difference; *UPDRS* Unified Parkinson's Disease Rating Scale

## Discussion

In this paper, we described an integrated protocol allowing reliable, safe MER during general anesthesia in STN DBS for PD, with a tangible impact on electrode positioning. Moreover, we put this finding in the context of the available literature through a systematic review. Importantly, this is the first work in which MERs were performed during asleep DBS using a frameless technique.

### Rationale of the work

Historically, STN targeting relied on atlases. The improvement in radiological techniques currently allow to identify the dorsolateral STN on preoperative MRI and to perform a direct targeting of STN [30, 35]. However, the majority of groups prefer to perform awake DBS surgery with the patient off medications, to record the typical STN discharge pattern through MER and to assess clinical improvement and absence of side effects through microstimulation. Nonetheless, the role of MER is debated [30], and its use has been associated with very rare but fearsome complications like hemorrhage and pneumocephalus [32, 35].

It has been traditionally deemed that MER cannot be recorded during asleep procedures [30], but evidence is rising that MER can be obtained also under general anesthesia [1, 3, 4, 5, 8, 9, 10, 11, 13, 15, 16, 17, 18, 19, 21, 22, 23, 25, 27, 28, 29, 32, 33, 35] (Table 3). Asleep surgery can be more comfortable for the patient, reducing fear and anxiety; several clinical factors can affect the choice to perform a general anesthesia. The opportunity to perform MER while the patient is comfortably asleep is a strong point favoring MER adoption during STN DBS.

### Findings from the case series and strong points

Our data confirm the feasibility and reliability of MER during general anesthesia (Fig. 1), adding evidence to the existing literature.

In the present work, we compared the planned electrode position with the actual one, finding out that MER led to a change in final positioning in the majority of cases (Table 1). Notably, in 23.8% cases, the central track was not satisfactory and other MER tracks were used. In a recent paper performing frame-based DBS [31], only 2% of electrodes were not placed in the central trace; on the other side, the same Authors acknowledge that in literature this percentage ranges from 26.5% to 68%. Importantly, this latter paper and other ones recorded only the change in MER track and not the final lead position on the central track. Finally, while direct STN targeting has reduced the need for MER [30], it is conceivable that the adoption of a frameless technique fosters the clinical role of intraoperative MER.

In our cohort, asleep STN DBS surgery for PD was effective in determining a significant improvement of UPDRS Part III score, both on- and off-medication, and a significant reduction of LEDD at 1-year follow-up (Table 2), in line with previously published data on clinical outcomes of STN DBS [7]. Given these data, we strongly recommend the use of MER during DBS intervention performed with general anesthesia, also considering that simple and effective techniques like MER are available with acceptable costs and risks and with no adjunctive stress for the patient.

### Limitations of the case series

Limitations of the study are its retrospective design and the lack of comparison of outcome of cases operated under general anesthesia with or without MER.

### Findings from the systematic literature review with metanalysis

Twenty-three papers were found in the literature describing the use of MER during asleep DBS. Taken together, these papers confirm feasibility and safety of MER in asleep patients. Several different anesthesiologic protocols have been described. Most authors, including our group, adopted a TIVA protocol based on propofol. Some modifications of the typical STN signal under propofol anesthesia have been described, including the absence of typical widening of the background baseline noise [13]. Of note, Myrov et al. systematically analyzed the difference in STN neuronal single-unit activity between awake and asleep state under propofol anesthesia [19]. They studied 25 parameters using machine-learning algorithms, finding significant differences between awake and asleep state in 14 of them. The most remarkable changes regarded the decrease in firing rate and the increase in bursting of neurons in asleep patients. However, no papers reported significant difficulties in recording STN MER signals using propofol anesthesia. The use of TCI and the monitoring of anesthesia level are key factors in allowing MER during TIVA with propofol [13, 21], as shown also in our series. Noteworthy, a non-negligible number of papers described successful MER under halogenate gases. In this setting, Fluchere et al. described a protocol for “controlled” general anesthesia using sevoflurane [8], while Chen et al. analyzed the different effect of sevoflurane and desflurane on MER [5]. Finally, Harries et al. reported no differences in MER quality between asleep patients operated under inhalation agents (nitrous oxide + isoflurane) or TIVA (propofol + remifentanil).

Most importantly, metanalysis confirmed the similar outcome of PD patients undergoing asleep DBS using MER as compared to awake DBS with MER (Tables 3 and 4 and Fig. 3). The trend towards a more pronounced LEDD reduction in asleep patients operated with MER reflects the finding by Park et al. [21] but should be interpreted with caution. In conclusion, based on our series and on literature review with metanalysis, nowadays MER during asleep DBS should be considered a technically straightforward procedure.

A future field of research could involve the assessment of the actual clinical impact of MER recording during asleep DBS. However, it is conceivable that a more accurate electrode placement is associated with an improved clinical benefit. It should be noted that a randomized clinical trial assessing this issue could be difficult to set up due to ethical concerns.

## Conclusions

MER is feasible and reliable during asleep STN DBS for PD, allowing to increase the accuracy of electrode positioning. We think that this technique is still valid in the era of direct STN targeting.

## Supplementary Information

Below is the link to the electronic supplementary material.

Supplementary file1 (PDF 108 KB)

## Data Availability

Source data are available from the Corresponding Author upon reasonable request.
