# Supplementary material for: Intraoperative microelectrode recording during asleep deep brain stimulation of subthalamic nucleus for Parkinson Disease. A case series with systematic review of the literature
Source: Neurosurg Rev. 2024 Jul 20;47(1):342. doi: 10.1007/s10143-024-02563-1 (PMC11271364; doi:10.1007/s10143-024-02563-1)
Supplement: Supplementary file 1 — Supplementary file1 (PDF 108 KB) [file 10143_2024_2563_MOESM1_ESM.pdf]

Intraoperative microelectrode recording during asleep deep brain stimulation of subthalamic nucleus for Parkinson Disease. A case series with systematic review of the literature. Alessandro Izzo et al; from the Departments of Neurosurgery, Neurology and Anesthesiology, Fondazione Policlinico Universitario Agostino Gemelli IRCCS, and the School of Medicine, Università Cattolica del Sacro Cuore, Rome, Italy. Corresponding author: Q. Giorgio D'Alessandris, Department of Neurosurgery, Fondazione Policlinico Universitario Agostino Gemelli IRCCS and School of Medicine, Università Cattolica del Sacro Cuore, Rome, Italy.
Journal: ***Neurosurgical Review***

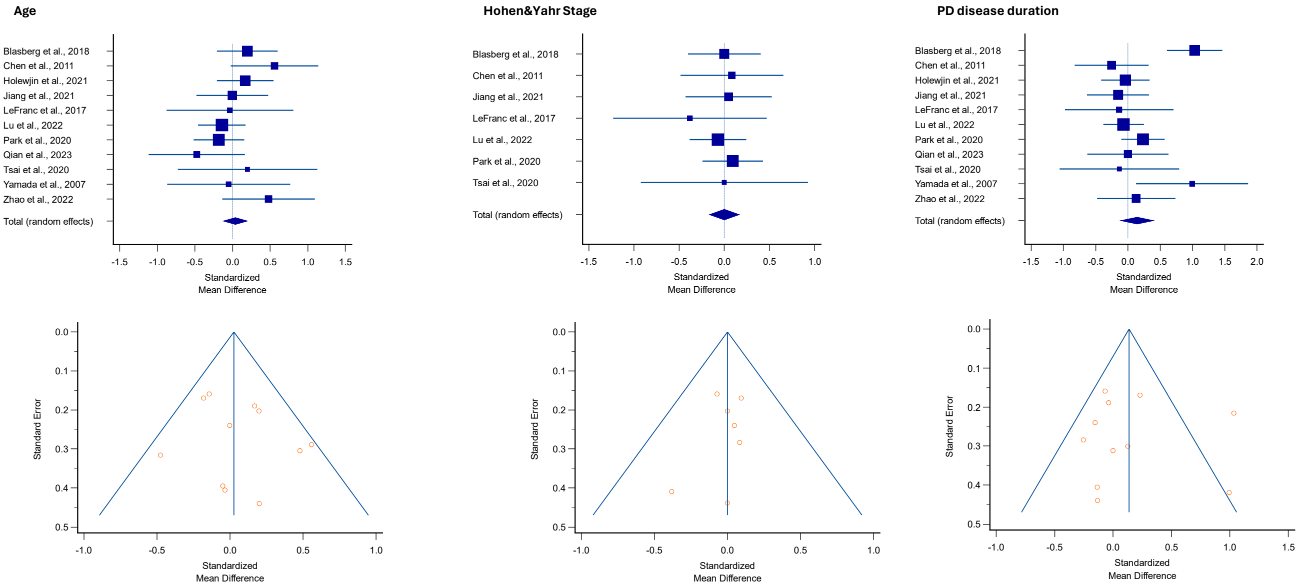

Supplementary Figure S1. Forest plots (upper row) and funnel plots (lower row) regarding age (left), Hohen&Yahr stage (center) and PD disease duration (right). In each forest plot, *right* favors asleep DBS and *left* favors awake DBS.
